# CDMOs Play a Critical Role in the Biopharmaceutical Ecosystem

**DOI:** 10.3389/fbioe.2022.841420

**Published:** 2022-03-21

**Authors:** Hideyuki Kurata, Tetsuya Ishino, Yasuhiro Ohshima, Masafumi Yohda

**Affiliations:** ^1^ Technology General Division, AGC Inc., Tokyo, Japan; ^2^ Institute of Engineering, Tokyo University of Agriculture and Technology, Tokyo, Japan; ^3^ AGC Biologics, Bothell, WA, United States; ^4^ Business Development Division, AGC Inc., Tokyo, Japan

**Keywords:** CDMO, biologics, bioprocess, monoclonal antibody, semiconductor, foundry

## Abstract

Biopharmaceutical industries have advanced significantly after the millennium. Novel biopharmaceuticals have been developed one after another, and blockbuster drugs have been produced. Accompanying the increase in the demand for biopharmaceuticals, a business model called “contract development manufacturing organization (CDMO)” has emerged. A CDMO is entrusted with the development and manufacturing of production processes from pharmaceutical companies. In this review, we identify the success factors of the biopharmaceutical CDMO by analyzing the foundry business for the semiconductor industry. Furthermore, we also review monoclonal antibody production platforms and new technologies that are critical aspects of differentiation strategies in the biopharmaceutical CDMO.

## Introduction

Biopharmaceutical drugs or biologics is a general term for drugs manufactured using biotechnology. Unlike small-molecule drugs that are chemically manufactured, biologics are complex molecules, such as proteins, or cells that are used as medicinal ingredients. Protein therapeutics was a niche market in the 20th century, but it has dramatically changed with the advent of antibody drugs in the 21st century. Eventually, protein therapeutics dominated the sales of new drugs in the pharmaceutical industry, resulting in paradigm shift (Ecker et al., 2015). The share of biologics in the total sales of the top 100 drugs was more than 16% in 2012 and expected to reach 55% by 2026. Global sales of biologics in prescription medicines continue to grow rapidly with an annual average growth rate of 9.6% from 2019 to 2026 (Evaluate Pharma World Preview 2020, 2021). Furthermore, new modalities, such as gene therapy drugs, have been developed to treat unmet medical needs.

**FIGURE 1:**
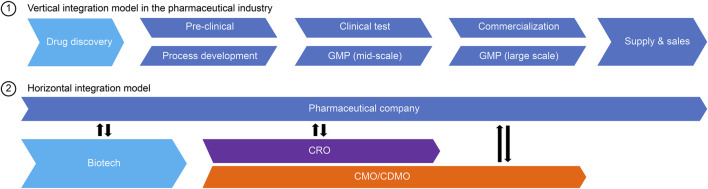
Horizontal division of roles in the pharmaceutical industry.

While the biopharmaceutical industry has made remarkable progress, the contract manufacturing and development organization (CDMO), which performs the development and manufacturing of drug substances in contract with biopharmaceutical companies, has grown simultaneously (Lakshmikanthan, 2007). Figure 1 shows an ecosystem of the pharmaceutical industry that is a horizontal division model of drug discovery by biotech and pharmaceutical companies as well as drug manufacture by CDMOs. The market size of the biopharmaceutical CDMO industry exceeded 10 billion US dollars in 2018. In addition to the continuous development of antibody drugs, various new biologics have emerged, expanding the biopharmaceutical CDMO market. The average annual growth rate is expected to be 10.2% from 2020 to 2025, which is higher than the small molecule drug market (8.0%) (BCC Research, 2020).

**FIGURE 2:**
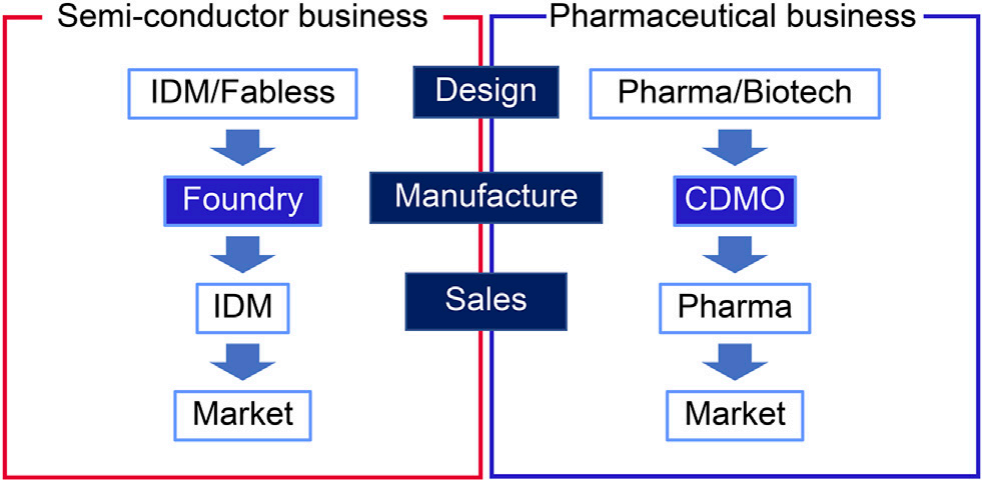
Comparison of semiconductor and pharmaceutical industries.

Prior to the biopharmaceutical industry, the semiconductor industry established a horizontal division model in the 1980s. There are three divisions in the semiconductor ecosystem as follows: fabless, foundries that manufacture microchips for other companies, and integrated device manufacturers (IDMs) (Anzenbacher and Wagner, 2020). Figure 2 shows a comparison of the horizontal business model between the pharmaceutical industry and the semiconductor industry. In this model, the positions of the design department of semiconductor companies and fabless companies correspond to the drug discovery department of pharmaceutical companies and drug discovery startups, respectively. Although each industry’s product is distinct, both industries suffer from the burdens of increasing costs of R&D for new products and manufacturing technology. The history of the development and technology platform of the semiconductor industry provides insights into the key success factors for the biopharmaceutical CDMO industry.

## Background of Semiconductor Foundry Growth

The historical background of the semiconductor industry has a significant influence on the growth of foundries. Before the rise of the foundry, fabless companies outsourced manufacturing to IDM. However, the growth of fabless was restricted by the manufacturing capacity and the lack of necessary manufacturing technologies for IDM. In the 1980s, many fabless companies started to enter the semiconductor business with the establishment of foundry companies. These fabless companies made a significant contribution to the innovation of semiconductor chips (Shalf, 2020). In the 2000s, fabless companies and other companies, such as Google and Amazon, started to design dedicated chips and outsourced manufacturing the chips at foundries. Due to the increasing demand for semiconductor manufacturing, the foundry market became 73.6 billion US dollars in 2018 and will be 151.2 billion US dollars in 2022. The compound annual growth rate is expected to be 11.6% by 2025 (IC Insights, 2021).

## Success Factors for Semiconductor Foundries

One of the key success factors of the foundry is to secure the supply of high-quality and high-performance semiconductor products in a timely manner. As the semiconductor market grows, foundries need great investments to ensure their manufacturing capacity. Nonetheless, IDM also requires significant capital investment for manufacturing equipment, but the operating ratio of the equipment depends on the sales of its new products. Therefore, a decrease in the operating ratio can be a management risk. In contrast, because foundries can receive orders of contracted manufacturing from both IDM and fabless companies, they can maintain the high operation ratio of the manufacturing equipment. Therefore, fabless companies can focus on design, and foundries can concentrate on securing manufacturing process capabilities (Liu, 2021). Currently, several strong foundries are leading the semiconductor industry. However, the coexistence of IDM, fabless, and foundries will be necessary for the advancement of the semiconductor industry (Hung et al., 2017).

Access to new technologies is another success factor for foundries. Microfabrication is one of the most critical technologies in the semiconductor manufacturing process. In the semiconductor industry, miniaturization of the devices proceeds according to Moore’s law (semiconductor integration rate doubles in 18–24 months). This evolution of microfabrication makes it possible to increase the number of transistors per unit area, significantly contributing to the increase in operating speed and reducing power consumption (Dennard et al., 1974). Advanced process technologies, such as extreme ultraviolet (EUV) light, have been invented to overcome microfabrication limits by conventional exposure equipment using ultraviolet light (Turkot et al., 2017; Shalf, 2020). In recent years, top-class foundries have quickly introduced these microfabrication technologies and developed chip manufacturing capabilities based on the latest design rules. As a result, the dependence on foundries with high manufacturing technology is increasing.

## Background of Growth of Biologics Contract Development Manufacturing Organizations

Pharmaceutical companies are also required to concentrate their investment and resources for new drug discovery (Blanco and Gardinier, 2020). The total R&D cost at mega-pharma (2006–2014) was in the range of 10–90 billion US dollars, and the number of new drugs launched tends to increase in proportion to the R&D cost (Schuhmacher et al., 2016). As a result, it is becoming difficult for pharmaceutical companies (equivalent to IDM in the semiconductor industry) to invest in process development and manufacturing as well as start to depend on CDMOs (equivalent to the foundry in the semiconductor industry) (Lakshmikanthan, 2007). Furthermore, due to the evolution of biotechnology, many innovative modalities for various rare diseases have been developed by drug discovery biotech (equivalent to fabless in the semiconductor industry) (O’Neil, 2014). Biotech as well as small and medium-sized pharmaceutical companies increasingly outsource manufacturing to avoid the risk of investing in production facilities.

## Success Factors for Biologics Contract Development Manufacturing Organizations

By comparing the semiconductor foundries and the biologics contract business, we found several important common factors for their success as follows:

1) Secure production technology using advanced technology.
2) Secure timely equipment capacity.
3) Appropriate service according to customer request.

In the semiconductor industry, manufacturing technology has been standardized with the development of manufacturing equipment, making it possible to outsource semiconductor manufacturing. Thus, fabless companies have grown in collaboration with foundries. In addition, semiconductor manufacturing technology has advanced significantly with microfabrication technology, making it difficult to create a product only by purchasing equipment. Foundries have advanced manufacturing technology by the combination of complicated processes and operating know-how. In the biopharmaceutical industry, manufacturing biologics, especially antibody drugs, have been well standardized. This standardization has made it easier to outsource the manufacture of biopharmaceutical drugs to CDMOs. In this context, the further development of biopharmaceutical CDMOs will depend on the advancement of their manufacturing technologies. In the following section, we review the production technology trends in antibody drugs, which are the mainstream biologics. We will also discuss the potential of next-generation manufacturing systems for biologics.

## Standardized Technology of Antibody Drug Production Boosts the Biopharmaceutical Contract Development Manufacturing Organization Business

An antibody is a protein used by the immune system to identify and neutralize foreign objects called antigens. Using the ability to bind various target molecules, many antibody drugs have been developed and put on the market (Lu et al., 2020). Antibodies consist of two polypeptide chains, a heavy (H) and a light (L) chain, each of which is composed of two regions, a constant region (C) and a variable region (V). The complementarity-determining region (CDR) of the variable region is essential to the ability of the antibody to bind to its intended target. Most antibody drugs are humanized antibodies that are constructed by transplanting the CDR from mouse monoclonal antibodies into human antibodies, indicating that antibody drugs share almost the same physical and chemical properties. Therefore, it is possible to standardize the manufacturing process of antibody drugs. The general flow of antibody drug manufacturing platforms is composed of upstream and downstream processes. The upstream process is comprised of processes of producing a cell line and culturing the cell line in a bioreactor, and the downstream process is comprised of processes of purifying the produced antibody component from culture media, inactivating the virus, and filling it as a pharmaceutical substance (Gronemeyer et al., 2014). Most of the processes are batch-type and are controlled by good manufacturing practice (GMP) production processes at pharmaceutical manufacturing sites. Because the antibody manufacturing process is standardized, it results in the standardization of manufacturing equipment, peripheral auxiliary materials, and raw material systems. The standardization of antibody manufacture has made it easier for biopharmaceutical companies to outsource their manufacturing to biopharmaceutical CDMOs (Lakhdar et al., 2007).

As mentioned in the previous chapter, the foundries of the semiconductor industry have established the position in which semiconductor manufacturing technology is equal to or better than the IDM by acquiring novel technology and accumulating proven track records of the manufacture of semiconductor devices. We believe that biopharmaceutical CDMOs can provide manufacturing technology of the same quality as pharmaceutical companies. To establish a similar position to the foundry in the semiconductor industry, it will be important for biopharmaceutical CDMOs to acquire and develop distinctive manufacturing technologies. In the following chapters, we will review recent trends in the production technology of antibody drugs while showing examples of advanced technology development at biopharmaceutical CDMOs.

## Cell Line Development Process

Various cell lines have been used to produce antibody drugs, but the most frequently used cell lines today are Chinese hamster ovary (CHO) cells. Although CHO cells have been used to produce various protein therapeutics in addition to antibody drugs, they are the most compatible production cells for antibody drugs in terms of high productivity, quality of glycosylation, and safety (Kunert and Reinhart, 2016). Examples of research and development on CHO cells performed by CDMOs are described below.

For the large-scale production of therapeutic proteins, including monoclonal antibodies (mAbs), recombinant Chinese hamster ovary (rCHO) cells are established using dihydrofolate reductase (DHFR)-based methotrexate (MTX) selection. However, it requires a multiround of MTX selection for stepwise gene amplification, resulting in a longer timeline for cell line generation (Porter et al., 2010). LONZA (Switzerland) has developed a CHO-GS (glutathione synthetase) system to resolve this problem. In the CHO-GS system, the selection of top-producing cell lines is based on controlling the balance between the expression level of GS and the concentration of its specific inhibitor, l-methionine sulfoximine (MSX). The CHO-GS system has attracted a wide range of customers from venture companies to major pharmaceutical companies because it requires only a single round of MSX selection. Recently, LONZA developed a method to introduce antibody genes site-specifically by homologous recombination in collaboration with Pfizer (Feary et al., 2021). Compared to the conventional method in which antibody genes are randomly inserted in the genome, the cells prepared by this method provide stable antibody expression levels. AGC Biologics (United States) also developed a CHEF1 system that significantly improves the productivity of recombinant proteins using the EF-1 alpha promoter (Running Deer and Allison, 2004). AGC Biologics further developed a system with higher antibody productivity, in which the DNA codon of the selection marker (dihydrofolate reductase) gene is modified to suppress the expression to amplify the introduced plasmid genes in the cell (Westwood et al., 2010).

As a recent trend, it is noteworthy that comprehensive analysis of metabolic pathways by omics analysis becomes essential for cell line development (Yusufi et al., 2017). For example, systematic analysis of typical CHO cell parent strains (CHO-K1, CHO-DXB11, and CHO-DG44) has shown that the sugar chain structure difference of the antibody is due to the difference in the expression levels of various genes involved (Lakshmanan et al., 2019). Omics analysis is also a powerful tool for optimizing media components. Ali et al. (2020) elucidated how the lack of cysteine in the medium reduces antibody productivity. Analysis of the transcriptome and proteome has led to the elucidation of the mechanism by which various factors, such as endoplasmic reticulum stress, affect antibody production. The omics analysis method is an effective method for improving production stocks. In addition, trials to enhance CHO cell antibody productivity by genome editing technologies, such as CRISPR/Cas9, are increasing (Ronda et al., 2014; Grav et al., 2017; Ley et al., 2019).

## Culture and Purification Process

Mass production of blockbuster marketed drugs was mainstream in the biopharmaceutical CDMO industry in the early 2000s. In recent years, however, the pipelines of biologics, such as rare diseases, have increased (Lu et al., 2020), which favors production schemes with a wider variety and smaller quantity. As a result, the demand for highly flexible single-use reactors has been increased (Jacquemart et al., 2016). Single-use equipment is disposable equipment made from plastic and is mainly used for the upstream process of biological production. Because each lot of production is prepared with new single-use equipment, cleaning and the accompanying validation are not required. Thus, production can be started faster than in stainless bioreactors. It has been demonstrated that there is no difference in product quality between single-use and stainless-steel reactors (Beck et al., 2020).

High-density culture or continuous culture methods have attracted attention as another technology in the upstream process to reduce capital costs and improve productivity (Jyothilekshmi and Jayaprakash, 2021). Continuous production allows for large quantities of culture in small facilities. Moreover, by continuous medium replacement, the concentrations of metabolites, such as lactic acid or ammonia, are kept low, increasing the lifetime of cells. Continuous culture may improve product quality. The perfusion process produces significantly lower bispecific antibody aggregates compared to the fed-batch process (Sinharoy et al., 2020).

By cell engineering and the optimization of the culture process, antibody productivity has significantly increased up to approximately 10 g/L culture. Accordingly, the downstream process has been optimized (Chahar et al., 2020). The general first step in antibody purification is affinity chromatography using resin-conjugating protein A, which binds to the antibody’s constant region. This step requires a large amount of the expensive protein A resin, raising production costs (Xenopoulos, 2015). The continuous chromatography method has attracted attention to solve this problem. Fedorenko et al. (2020) developed continuous countercurrent tangential chromatography (CCTC), which efficiently purifies antibodies with a smaller amount of column resin. In this method, protein A resin is circulated to continuously adsorb and desorb antibodies; therefore, the necessary resin amount is approximately 3 times smaller than that of the batch method, while the product quality (HCP, DNA, and single molecular weight fraction) of the antibody purified by the CCTC method is equivalent to that of the batch method. Although the development of alternative resins has been reported (Ghose et al., 2006; Ishihara et al., 2018), it is still uncertain whether they can replace protein A resin in the manufacture of antibody drugs.

## Next-Generation Manufacturing Process

**FIGURE 3:**
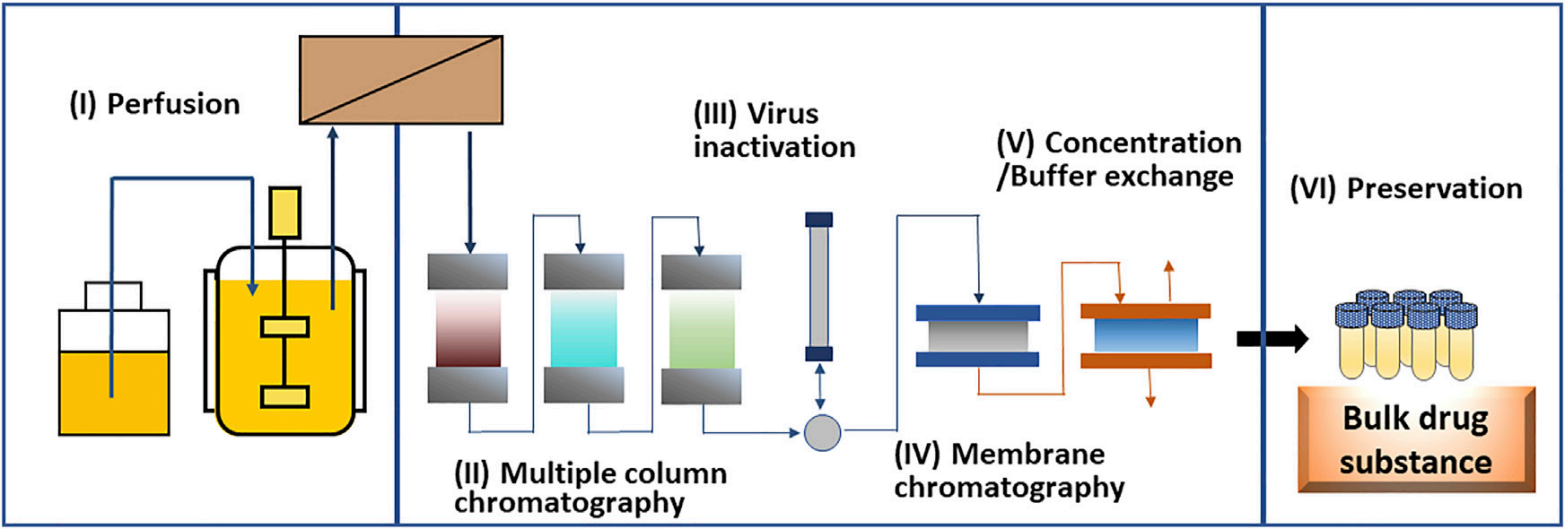
Schematic drawing of an example of end-to-end continuous manufacturing process for therapeutic monoclonal antibody.

The end-to-end continuous manufacturing process draws attention as a favorable biologics manufacturing system in the future. Figure 3 depicts an example of continuous process of therapeutic antibody production with (I) perfusion bioreactor, (II) multiple column chromatography capture, (III) virus inactivation, (IV) membrane chromatography polishing, (V) concentration/buffer exchange, and (VI) preservation processes. The continuous process does not require the extraction of products for each process, which could reduce the total time of manufacturing and the number of operators as well as the risk of human error and contamination (Khanal and Lenhoff, 2021). Furthermore, highly reliable quality assurance may be achieved by monitoring the quality in real time (Walther et al., 2015). Some cost simulations show that the manufacturing cost of antibody drugs will be reduced by continuous production (Pollock et al., 2017; Arnold et al., 2019). Although continuous manufacturing processes produce various small molecule drugs, the continuous production of biologics is still under development. A complete continuous production process of antibody drugs on the laboratory scale has been reported (Steinebach et al., 2017). Using this method, continuous culture was successfully performed for 18 days and the product quality was acceptable compared to the batch method.

Despite the advantages as discussed above, progress to date in implementing continuous manufacturing appears to be slower than anticipated. This is mainly because development and implementation of continuous processes require long period of time to recoup an initial investment. Badman et al. have proposed some interesting idea, for instance such as tax and regulatory incentives, to promote the transition from batch to continuous systems (Badman et al., 2019). Use of the continuous production method is also limited due to the high complexity of the system. Although digital technologies, such as supervisory control and data acquisition (SCADA), have also been developed (Feidl et al., 2020), there will be room for improvement to achieve the end-to-end continuous manufacturing of biologics in actual settings. Process analytical technologies (PAT) has been developed in batch or fed-batch operations (Maruthamuthu et al., 2020). We believe that further development of PAT combined with digital data management systems will also help transition to the continuous production in biopharmaceutical industries (Eifert et al., 2020).

## Conclusion

In the biopharmaceutical industry, many technological innovations have occurred both in drug discovery and the manufacturing process. At the same time, the horizontal division model in the pharmaceutical industry has become essential; biopharmaceutical companies (and biotech companies) focus more on drug discovery and development, while CDMOs focus more on process development and manufacture. Biopharmaceutical CDMOs could learn from the semiconductor industry, in which the foundries established a technical status similar to that of the IDM. It will be more critical for CDMOs to invest more in technological development and acquire new technology to maintain a competitive edge. We believe that CDMOs will contribute significantly to a healthy life by continuous support to the biopharmaceutical industry in producing biopharmaceuticals for more diseases.

## References

[B1] Ali A. S. Chen R. Raju R. Kshirsagar R. Gilbert A. Zang L. (2020). Multi-Omics Reveals Impact of Cysteine Feed Concentration and Resulting Redox Imbalance on Cellular Energy Metabolism and Specific Productivity in CHO Cell Bioprocessing. Biotechnol. J. 15, 1900565. 10.1002/biot.201900565 PMC7880547 32170810

[B2] Anzenbacher A. Wagner M. (2020). The Role of Exploration and Exploitation for Innovation success: Effects of Business Models on Organizational Ambidexterity in the Semiconductor Industry. Int. Entrep. Manag. J. 16, 571–594. 10.1007/s11365-019-00604-6

[B3] Arnold L. Lee K. Rucker-Pezzini J. Lee J. H. (2019). Implementation of Fully Integrated Continuous Antibody Processing: Effects on Productivity and COGm. Biotechnol. J. 14, 1800061. 10.1002/biot.201800061 29729129

[B4] Badman C. Cooney C. L. Florence A. Konstantinov K. Krumme M. Mascia S. (2019). Why We Need Continuous Pharmaceutical Manufacturing and How to Make it Happen. J. Pharm. Sci. 108, 3521–3523. 10.1016/j.xphs.2019.07.016 31381905

[B5] BCC Research (2020). Global Market Opportunities and Competitive Landscape for CDMO. Wellesley: BCC Research LLC.

[B6] Beck C. Werz S. Herrmann A. Meiringer C. T. A. (2020). Comparative Study for the Production of Monoclonal Antibodies in Single-Use vs Stainless Steel Bioreactors Based on Product Quality and Stress Factor. Eng. Rep. 2, e12197. 10.1002/eng2.12197

[B7] Blanco M.-J. Gardinier K. M. (2020). New Chemical Modalities and Strategic Thinking in Early Drug Discovery. ACS Med. Chem. Lett. 11, 228–231. 10.1021/acsmedchemlett.9b00582 32184948 PMC7073867

[B8] Chahar D. S. Ravindran S. Pisal S. S. (2020). Monoclonal Antibody Purification and its Progression to Commercial Scale. Biologicals 63, 1–13. 10.1016/j.biologicals.2019.09.007 31558429

[B9] Dennard R. H. Gaensslen F. H. Yu H.-N. Rideout V. L. Bassous E. LeBlanc A. R. (1974). Design of Ion-Implanted MOSFET's with Very Small Physical Dimensions. IEEE J. Solid-state Circuits 9, 256–268. 10.1109/JSSC.1974.1050511

[B10] Ecker D. M. Jones S. D. Levine H. L. (2015). The Therapeutic Monoclonal Antibody Market. MAbs 7, 9–14. 10.4161/19420862.2015.989042 25529996 PMC4622599

[B11] Eifert T. Eisen K. Maiwald M. Herwig C. (2020). Current and Future Requirements to Industrial Analytical Infrastructure-Part 2: Smart Sensors. Anal. Bioanal. Chem. 412, 2037–2045. 10.1007/s00216-020-02421-1 32055909 PMC7072042

[B12] Evaluate Pharma World Preview 2020 (2021). Outlook to 2026. Available from: https://www.evaluate.com/thought-leadership/pharma/evaluatepharma-world-preview-2020-outlook-2026 (Accessed November 26, 2021).

[B13] Feary M. Moffat M. A. Casperson G. F. Allen M. J. Young R. J. (2021). CHOK1SV GS-KO SSI Expression System: A Combination of the Fer1L4 Locus and Glutamine Synthetase Selection. Biotechnol. Prog. 37, e3137. 10.1002/btpr.3137 33609084

[B14] Fedorenko D. Dutta A. K. Tan J. Walko J. Brower M. Pinto N. D. S. (2020). Improved Protein A Resin for Antibody Capture in a Continuous Countercurrent Tangential Chromatography System. Biotechnol. Bioeng. 117, 646–653. 10.1002/bit.27232 31784975

[B15] Feidl F. Vogg S. Wolf M. Podobnik M. Ruggeri C. Ulmer N. (2020). Process-wide Control and Automation of an Integrated Continuous Manufacturing Platform for Antibodies. Biotechnol. Bioeng. 117, 1367–1380. 10.1002/bit.27296 32022243

[B16] Ghose S. Hubbard B. Cramer S. M. (2006). Evaluation and Comparison of Alternatives to Protein A Chromatography. J. Chromatogr. A 1122, 144–152. 10.1016/j.chroma.2006.04.083 16750212

[B17] Grav L. M. la Cour Karottki K. J. Lee J. S. Kildegaard H. F. (2017). Application of CRISPR/Cas9 Genome Editing to Improve Recombinant Protein Production in CHO Cells. Methods Mol. Biol. 1603, 101–118. 10.1007/978-1-4939-6972-2_7 28493126

[B18] Gronemeyer P. Ditz R. Strube J. (2014). Trends in Upstream and Downstream Process Development for Antibody Manufacturing. Bioengineering 1, 188–212. 10.3390/bioengineering1040188 28955024

[B19] Hung H.-C. Chiu Y.-C. Wu M.-C. (2017). Analysis of Competition between IDM and Fabless-Foundry Business Models in the Semiconductor Industry. IEEE Trans. Semicond. Manufact. 30, 254–260. 10.1109/TSM.2017.2699739

[B20] IC Insights (2021). Foundry Market Tracking toward Record-Tying 23% Growth in 2021. Scottsdale, Arizona USA: IC Insights Research Bulletin.

[B21] Ishihara T. Miyahara M. Yamamoto K. (2018). Monoclonal Antibody Purification Using Activated Carbon as a Replacement for Protein A Affinity Chromatography. J. Chromatogr. B 1102-1103, 1–7. 10.1016/j.jchromb.2018.10.004 30366207

[B22] Jacquemart R. Vandersluis M. Zhao M. Sukhija K. Sidhu N. Stout J. (2016). A Single-Use Strategy to Enable Manufacturing of Affordable Biologics. Comput. Struct. Biotechnol. J. 14, 309–318. 10.1016/j.csbj.2016.06.007 27570613 PMC4990569

[B23] Jyothilekshmi I. Jayaprakash N. S. (2021). Trends in Monoclonal Antibody Production Using Various Bioreactor Syst. J. Microbiol. Biotechnol. 31, 349–357. 10.4014/jmb.1911.11066 32238761 PMC9705917

[B24] Khanal O. Lenhoff A. M. (2021). Developments and Opportunities in Continuous Biopharmaceutical Manufacturing. MAbs 13, 1903664. 10.1080/19420862.2021.1903664 33843449 PMC8043180

[B25] Kunert R. Reinhart D. (2016). Advances in Recombinant Antibody Manufacturing. Appl. Microbiol. Biotechnol. 100, 3451–3461. 10.1007/s00253-016-7388-9 26936774 PMC4803805

[B26] Lakhdar K. Savery J. Papageorgiou L. G. Farid S. S. (2007). Multiobjective Long-Term Planning of Biopharmaceutical Manufacturing Facilities. Biotechnol. Prog. 23, 1383–1393. 10.1021/bp0701362 17924645

[B27] Lakshmanan M. Kok Y. J. Lee A. P. Kyriakopoulos S. Lim H. L. Teo G. (2019). Multi-omics Profiling of CHO Parental Hosts Reveals Cell Line-specific Variations in Bioprocessing Traits. Biotechnol. Bioeng. 116, 2117–2129. 10.1002/bit.27014 31066037

[B28] Lakshmikanthan J. (2007). Outsourcing: Biologics Manufacturing: The CMO Advantage. Available from: https://www.biopharminternational.com/view/outsourcing-biologics-manufacturing-cmo-advantage (Accessed December 17, 2021).

[B29] Ley D. Pereira S. Pedersen L. E. Arnsdorf J. Hefzi H. Davy A. M. (2019). Reprogramming AA Catabolism in CHO Cells with CRISPR/Cas9 Genome Editing Improves Cell Growth and Reduces Byproduct Secretion. Metab. Eng. 56, 120–129. 10.1016/j.ymben.2019.09.005 31526854

[B30] Liu M. (2021). Taiwan and the Foundry Model. Nat. Electron. 4, 318–320. 10.1038/s41928-021-00576-y

[B31] Lu R.-M. Hwang Y.-C. Liu I.-J. Lee C.-C. Tsai H.-Z. Li H.-J. (2020). Development of Therapeutic Antibodies for the Treatment of Diseases. J. Biomed. Sci. 27, 1–30. 10.1186/s12929-019-0592-z 31894001 PMC6939334

[B32] Maruthamuthu M. K. Rudge S. R. Ardekani A. M. Ladisch M. R. Verma M. S. (2020). Process Analytical Technologies and Data Analytics for the Manufacture of Monoclonal Antibodies. Trends Biotechnol. 38, 1169–1186. 10.1016/j.tibtech.2020.07.004 32839030 PMC7442002

[B33] O’Neil D. A. (2014). A Better Fit? Biotech versus Big Pharma in Orphan/rare Disease Drug Research. Expert Opin. Orphan Drugs 2, 317–319. 10.1517/21678707.2014.900433

[B34] Pollock J. Coffman J. Ho S. V. Farid S. S. (2017). Integrated Continuous Bioprocessing: Economic, Operational, and Environmental Feasibility for Clinical and Commercial Antibody Manufacture. Biotechnol. Prog. 33, 854–866. 10.1002/btpr.2492 28480535 PMC5575510

[B35] Porter A. J. Dickson A. J. Racher A. J. (2010). Strategies for Selecting Recombinant CHO Cell Lines for cGMP Manufacturing: Realizing the Potential in Bioreactors. Biotechnol. Prog. 26, 1446–1454. 10.1002/btpr.442 20623581

[B36] Ronda C. Pedersen L. E. Hansen H. G. Kallehauge T. B. Betenbaugh M. J. Nielsen A. T. (2014). Accelerating Genome Editing in CHO Cells Using CRISPR Cas9 and CRISPy, a Web-Based Target Finding Tool. Biotechnol. Bioeng. 111, 1604–1616. 10.1002/bit.25233 24827782 PMC4312910

[B37] RunningDeer J. Allison D. S. (2004). High-Level Expression of Proteins in Mammalian Cells Using Transcription Regulatory Sequences from the Chinese Hamster EF-1α Gene. Biotechnol. Prog. 20, 880–889. 10.1021/bp034383r 15176895

[B38] Schuhmacher A. Gassmann O. Hinder M. (2016). Changing R&D Models in Research-Based Pharmaceutical Companies. J. Transl. Med. 14, 105–116. 10.1186/s12967-016-0838-4 27118048 PMC4847363

[B39] Shalf J. (2020). The Future of Computing beyond Moore's Law. Phil. Trans. R. Soc. A. 378, 20190061. 10.1098/rsta.2019.0061 31955683

[B40] Sinharoy P. Aziz A. H. Majewska N. I. Ahuja S. Handlogten M. W. (2020). Perfusion Reduces Bispecific Antibody Aggregation via Mitigating Mitochondrial Dysfunction-Induced Glutathione Oxidation and ER Stress in CHO Cells. Sci. Rep. 10, 16620. 10.1038/s41598-020-73573-4 33024175 PMC7538420

[B41] Steinebach F. Ulmer N. Wolf M. Decker L. Schneider V. Wälchli R. (2017). Design and Operation of a Continuous Integrated Monoclonal Antibody Production Process. Biotechnol. Prog. 33, 1303–1313. 10.1002/btpr.2522 28691347

[B42] Turkot B. Carson S. Lio A. (2017). “Continuing Moore's Law with EUV Lithography,” in Proceeding of the 2017 IEEE International Electron Devices Meeting (IEDM), San Francisco, CA, USA, Dec. 2017 (IEEE), 1441–1443. 10.1109/iedm.2017.8268390

[B43] Walther J. Godawat R. Hwang C. Abe Y. Sinclair A. Konstantinov K. (2015). The Business Impact of an Integrated Continuous Biomanufacturing Platform for Recombinant Protein Production. J. Biotechnol. 213, 3–12. 10.1016/j.jbiotec.2015.05.010 26014522

[B44] Westwood A. D. Rowe D. A. Clarke H. R. G. (2010). Improved Recombinant Protein Yield Using a Codon Deoptimized DHFR Selectable Marker in a CHEF1 Expression Plasmid. Biotechnol. Prog. 26, 1558–1566. 10.1002/btpr.491 20949444

[B45] Xenopoulos A. (2015). A New, Integrated, Continuous Purification Process Template for Monoclonal Antibodies: Process Modeling and Cost of Goods Studies. J. Biotechnol. 213, 42–53. 10.1016/j.jbiotec.2015.04.020 25959171

[B46] Yusufi F. N. K. Lakshmanan M. Ho Y. S. Loo B. L. W. Ariyaratne P. Yang Y. (2017). Mammalian Systems Biotechnology Reveals Global Cellular Adaptations in a Recombinant CHO Cell Line. Cel Syst. 4, 530–542. e6. 10.1016/j.cels.2017.04.009 28544881

